# Cancer mortality patterns in Ghana: a 10-year review of autopsies and hospital mortality

**DOI:** 10.1186/1471-2458-6-159

**Published:** 2006-06-20

**Authors:** Edwin K Wiredu, Henry B Armah

**Affiliations:** 1 Department of Pathology, University of Ghana Medical School, College of Health Sciences, University of Ghana, Korle-Bu, Accra, Ghana; 2 School of Allied Health Sciences, College of Health Sciences, University of Ghana, Korle-Bu, Accra, Ghana

## Abstract

**Background:**

Cancer mortality pattern in Ghana has not been reviewed since 1953, and there are no population-based data available for cancer morbidity and mortality patterns in Ghana due to the absence of a population-based cancer registry anywhere in the country.

**Methods:**

A retrospective review of autopsy records of Department of Pathology, and medical certificate of cause of death books from all the wards of the Korle-Bu Teaching Hospital (KBTH), Accra, Ghana during the 10-year period 1991–2000 was done.

**Results:**

The present study reviews 3659 cancer deaths at the KBTH over the 10-year period. The male-to-female ratio was 1.2:1. The mean age for females was 46.5 [Standard Deviation (SD), 20.8] years, whilst that of males was 47.8 (SD, 22.2) years. The median age was 48 years for females and 50 years for males.Both sexes showed a first peak in childhood, a drop in adolescence and young adulthood, and a second peak in the middle ages followed by a fall in the elderly, with the second peak occurring a decade earlier in females than in males. The commonest cause of cancer death in females was malignancies of the breast [Age-Standardized Cancer Ratio (ASCAR), 17.24%], followed closely by haematopoietic organs (14.69%), liver (10.97%) and cervix (8.47%). Whilst in males, the highest mortality was from the liver (21.15%), followed by prostate (17.35%), haematopoietic organs (15.57%), and stomach (7.26%).

**Conclusion:**

Considering the little information available on cancer patterns in Ghana, this combined autopsy and death certification data from the largest tertiary hospital is of considerable value in providing reliable information on the cancer patterns in Ghana.

## Background

Knowledge of cancer patterns in Africa is woefully inadequate [1-7], and population-based epidemiological data on the occurrence of cancer in sub-Saharan Africa, especially, are sparse. Until recently, cancers and other non-communicable diseases were thought to be unimportant public health problems in developing countries, like Ghana, because of the overwhelming high prevalence of communicable diseases. The previous studies have focused on cancer morbidity data from the few established cancer registries in Africa, with very few reporting on cancer mortality data.

Malignant neoplasms accounted for 914 (2.6%) of all 34,598 admissions, and 141 (5.6%) of all 2,501 deaths at the Korle-Bu Teaching Hospital (KBTH) in the year 1996[8]. The case fatality risk from malignancies was thus 15.4%[8]. A review of the cancer register in the Department of Child Health, KBTH, over a 40-month period revealed that malignancies accounted for 1.67% of all admissions, with lymphomas (mainly Burkitt's lymphomas) being the commonest tumour (67%),followed by retinoblastoma (8.6%), leukaemia (8.2%) and Wilm's tumour (7.8%)[9]. The above cancer morbidity trend in the Ghanaian paediatric population is similar to the picture seen in West Africa and other developing African countries [9-22], with the exception of Namibia[23].

Previous reviews of cancer in Ghana have been mainly restricted to the study of single cancers, rather than the relative contributions of the various cancers to the disease burden because of the absence of a population-based registry. One of such reviews of autopsy material at the KBTH over the 10-year period from 1972–1981 (two decades earlier than this study), with emphasis on carcinoma of the pancreas, revealed that pancreatic cancers accounted for 5.8% of all cancer deaths over that period[24]. There has been no recent report on a systematic study of cancer mortality pattern across all age groups in Ghana. The problems of collecting cancer mortality statistics in developing countries have been described [1-7]. Knowledge of cancer patterns in Africa have largely been based on hospital series collected by clinicians and pathologists [1-7]. Despite the problems associated with interpreting data from hospital-based series, they are an invaluable source of information on cancer patterns where incidence and mortality data are unavailable. Ghana is on the threshold of starting a population-based cancer registry that will collect data on both morbidity and mortality. It is therefore an opportune time to attempt to present information on cancer mortality pattern before the advent of the registry to serve as the basis for future reference. This paper is partly hospital-based data and partly community-derived data to try and present as close a picture to the true situation as is possible now. This study was therefore carried out to establish the relative contributions of the various cancers to the cancer deaths certified and/or autopsied at the Korle-Bu Teaching Hospital, Accra, Ghana over a period of 10-years (1991–2000).

## Methods

All cancer deaths diagnosed at autopsy in the 10-year period from the beginning of January 1991 to the end of December 2000 were retrieved from the autopsy logbooks of the Department of Pathology, and all certified cancer deaths for which medical certificates of cause of death were issued in all the wards and units of the Korle-Bu Teaching Hospital over the same period were also collected and reviewed retrospectively to find all cancer deaths seen from 1991 to 2000 inclusive. All cases were entered into a database using the Paradox for workgroups microcomputer software (Boreland International, Inc) and later transferred into the EPI-INFO microcomputer software version 3.3 (Centres for Disease Control, Atlanta, USA.). Three thousand, six hundred and fifty-nine (3659) cases were retrieved and reviewed. Information collected on each case included the name, age, sex, date of death and the site of the primary tumour. To avoid double enumeration of autopsied cases for which certificates of cause of death were subsequently issued in the hospital, the database programme was set up to reject any case that had the same name, age, sex and site as an already registered case until this had been proved to be an entirely new case.

The primary site and morphology data were coded using the International Classification of Diseases (10^th ^Edition, ICD-10, World Health Organization)[25]. The ages were grouped according to the format used by International Agency for Research on Cancer (IARC, Lyon, France) for cancer reporting [1-7], namely 0–14, 15–24, 25–34, 35–44, 45–54, 55–64, and ≥65 years. Cases were analyzed according to sex and age distribution for all cases, and sex and age distribution for selected sites. The relative frequency (RF)of different cancer types varies greatly in relation to the age group studied, and thus the proportion of different cancers in a particular series is strongly influenced by the age composition of the series. This poses a problem when comparing summary statistics derived from populations of different age structures[1]. To overcome this problem and allow for comparison of proportions derived from this study with those derived from case series of different age structures, the age-standardized cancer ratio (ASCAR) was chosen as the standardization method. The reference case standard distribution used to calculate ASCAR was the standard age distribution of cancer cases for developing countries, namely 0–14 (5%), 15–24 (5%), 25–34 (5%), 35–44 (10%), 45–54 (20%), 55–64 (25%), and ≥ 65 (30%) years[1]. Tumours of the skin were excluded in calculating the ASCARaccording to convention [1-7]. The protocol for the study was approved by the Ethical and Protocol Review Committee of the University of Ghana Medical School, Accra, Ghana.

## Results

There were 1651 (45.1%) females and 2008 (54.9%) males in the series, making a total of 3659 cases. The male to female ratio is thus 1.2:1. The mean age for females was 46.5 years (SD, 20.8) and that of males was 47.8 years (SD, 22.2). The median age was 48 years for females and 50 years for males. The ages were not stated for 1 female and 4 males. Both sexes showed a peak in childhood with a drop in adolescence and young adulthood, and a rise to another peak in the middle ages followed by a fall in the elderly. The second peak occurs a decade earlier in females than in males.

The number of cases from each primary cancer site by age group, the summary RFrates of these sites and the ASCAR in females and males are shown in Tables 1 and 2, respectively. In females, malignancies of the breast (ASCAR, 17.24%) are the commonest cause of deaths, followed by the haematopoietic organs (lymphomas and leukaemias, 14.69%), and then the liver (10.97%), and the cervix (8.47%). In males, the highest mortality is from the liver (ASCAR, 21.15%), followed by the prostate (17.35%), and the haematopoietic organs (15.57%), and the stomach (7.26%). Deaths from tumours of the anus and eyes are uncommon in both sexes, while deaths from laryngeal cancers in female (ASCAR, 0.1%) are much less common than in males (2.75%), and deaths from stomach cancers in males (7.26%) are much more common than in females (4.76%).

**Figure 1:**
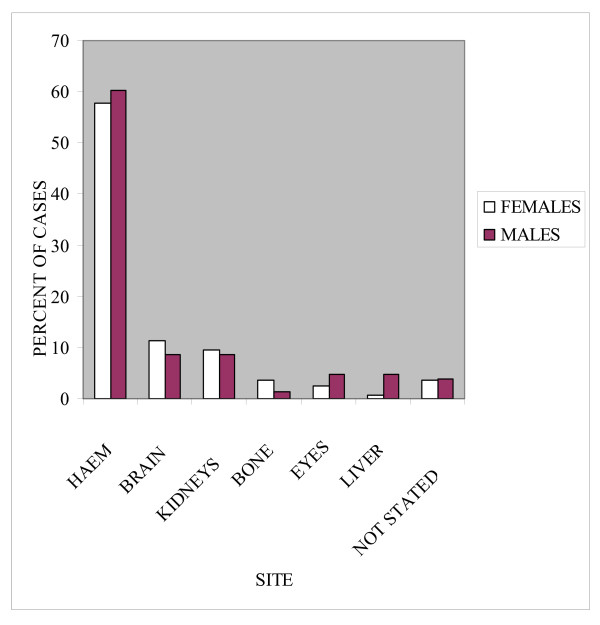
Gender distribution of cases in children.

There were 375 children (< 15 years), of which 209 (55.7%) were males and 166 (44.3%) were females, giving a paediatric male to female ratio of 1.26:1. Figure 1 shows the gender distribution of the various sites involved by tumours in children. In both sexes, the malignancies with the highest mortalityare those of the haematopoietic system, 57.8% of all paediatric cases in females and 60.3% among males. These are followed by brain and renal tumours. Liver and eye tumours feature in the top six death-causing malignancies in male children, but appear to be of less importance among female children, while bone tumours seem more important in female than male children.

The number of females in the adolescent and young adult age group (15–24 years) was 106 (42.7%), while males were 142 in number (57.3%), giving a male to female ratio of 1.3:1. Again, malignancies of the haematopoietic system dominate the picture, causing 43.4% of cancer deaths in females and 50.7% of the male cancer deaths. These are followed in the females by brain and then hepatic followed by ovarian tumours. In the males, haematopoietic malignancies were followed by hepatic, bone and brain tumours in that order. While in the children, bone tumours appeared more important as a cause of death in females than in males, in the adolescents and young adults the converse is found. Tumours of the eye continue to be more important in males than in female adolescents and young adults, as they were in children. An interesting finding is the occurrence of deaths due to endometrial tumours in this young age group, all of which were found to be due to choriocarcinoma, and the RF of mortality from pancreatic tumours of 4.2% in males and 0% in females. In this series, these pancreatic tumours occurred mainly in young adults (mean age 20 years). Bladder cancers contributed to 1.4% of cancer deaths in male adolescents and young adults, but 0% in females of the same age group. Conversely, soft tissue cancers contributed to 2.8% of cancer deaths in female adolescents and young adults, but 0% in males of the same age group.

The gender distribution of the RF of mortality from the various primary cancer sites in older adults, including the elderly, showed that the commonest fatal malignancies in females are those arising in the breast, while in males hepatic tumours are the commonest. Other gender differences in the pattern of cancer mortality in older adults that clearly stand out include laryngeal tumours which are about 27 times, oesophageal cancers which are about 6 times, and liver cancers which are about twice as common in males as in females. Additionally, lung and bladder cancers are about twice as common in male than female older adults. Gallbladder cancers are 5 times commoner in female than male older adults. The RF of pancreatic cancer deaths are similar between male and female older adults, in contrast to the 4.2% relative frequency in male adolescents and young adults and 0% in females of that age group.

## Discussion

Cancer is a worldwide public health problem. It accounts for 12.5% of all deaths, more than the percentage of deaths caused by HIV/AIDS, tuberculosis and malaria put together, is the second leading cause of death in developed countries, and is among the three leading causes of death for adults in developing countries[26]. In 2002, there were 6.7 million cancer deaths worldwide with less than 5% of these in sub-Saharan Africa. It is estimated that by 2020, cancer could kill 10.3 million people worldwide, with a 50 to 75 percentage increase in cancer mortality in sub-Saharan Africa[26]. This is the first report of cancer mortality patterns in Ghana, since the report of Edington in 1953[27]. The data used in the study were collected from two sources, namely medical certificates of cause of death issued in the hospital for deaths occurring in the hospital and autopsy records of the Department of Pathology of the hospital. Information from these sources was combined in an attempt to overcome some of the bias inherent in using data from either source. The Department of Pathology carries out an average of 5,000 autopsies annually, the vast majority (75%) of which are referred for autopsy by the coroner. Such coroner-referred deaths normally occur in the community (outside any hospital facility) or in the hospital usually in less than 24 hours of admission, when a firm cause of death would not have been determined. However, all hospital-based data on cancer are biased by selective factors that operate in hospitals[1]. The hospital may not comprehensively cover the population of interest. Thus hospital data may not completely reflect what happens at the community level as comparisons based on relative frequency of different cancers in hospital-based series will be biased in terms of the probability that the different cancers occurring in the community will be included.

Cancers are emerging as a significant health problem in several parts of Africa[8,9,13-18], though infections remain the leading cause of morbidity and mortality. Indeed, cancers accounted for 2.6% of all admissions, and 5.6% of all deaths at the KBTH in the year 1996 [8]. In the current study, malignancies of the breast were the leading cause of deaths in females, followed closely by the haematopoietic organs, liver and the cervix. In males, the highest mortality was from the liver, followed closely by the prostate, the haematopoietic organs and the stomach. The relatively high position of liver, haematopoietic and pancreas cancers in both sexes may be partly as a result of their high case mortality rate in Ghana. A recent review of cancer morbidity in adults in Ibadan, Nigeria showed prostate cancer as the most common cancer in males, whilst breast cancer topped the list in females[28]. The first such review in the same population reported that cancers of the reticuloendothelial system topped the list[29], while the second observed that liver cancer was commonest among males and cervical cancer was commonest among females[30]. Cancer mortality figures can be used as a good approximation of incidence of cancers for which mortality is high, such as hepatocellular carcinoma. Cancers with high survival rates will be underrepresented in such series. Hospital autopsy series, although having good diagnostic information, suffer from selective factors that operate in causing admission to a hospital and in having an autopsy. Thus they are not representative of deaths in a population[1].

The International Agency for Research on Cancer (IARC) has recently published GLOBOCAN 2002 estimates of cancer incidence, mortality and prevalence for Ghana, with the acknowledgement that the degree of detail and quality of this source of data (both the accuracy of the recorded cause of death and the completeness of the registration) vary considerably[26]. The GLOBOCAN 2002 estimates for Ghana are therefore a mixture of real data, extrapolations from limited samples, and informed guesses[26]. In contrast, this series is based on only actual data and is the best contemporary information on the cancer mortality pattern in Ghana, though it is not derived from a nationally-based cancer registry and therefore may not be representative of the national cancer mortality pattern.

GLOBOCAN 2002[26] reported that the top 10 causes of cancer mortality in descending order in females in Ghana were cervix, breast, liver, haematopoietic organs, stomach, colorectal, ovary, bladder, with pancreas and Kaposi sarcoma tied for the 9^th ^and 10^th ^position. In this series, the top 10 causes of cancer mortality in descending order in females in Ghana were breast, haematopoietic organs, liver, cervix, ovary, pancreas, stomach, colorectal, gall bladder and brain. Therefore, both GLOBOCAN and this series concur that the top 4 causes of cancer deaths in Ghanaian females are undoubtedly breast, cervix, haematopoietic organs and liver, though cervix in 1^st ^position for GLOBOCAN was 4^th ^in this series and haematopoietic organs in 2^nd ^position in this series was in 4^th ^position for GLOBOCAN for Ghanaian females.

GLOBOCAN 2002 [26]reported that the top 10 causes of cancer mortality in descending order in males in Ghana were prostate, liver, haematopoietic organs, colorectal, bladder, Kaposi sarcoma, stomach, lung, larynx and oral cavity. In this series, the top 10 causes of cancer mortality in descending order in males in Ghana were liver, prostate, haematopoietic organs, stomach, pancreas, bladder, lung, colorectal, brain and larynx. Therefore, both GLOBOCAN and this series concur that the top 3 causes of cancer deaths in Ghanaian males are undoubtedly liver, prostate and haematopoietic organs, with haematopoietic cancers in 3^rd ^position for both, and liver and prostate cancers swapping positions in the two series for Ghanaian males.

In both sexes of Ghanaian children combined, themalignancies with the highest mortality are those of the haematopoietic system, followed by brain, kidney, eyes, liver and bone tumours in that order. This cancer mortality picture is comparable to the reported cancer morbidity trend in the Ghanaian paediatric population[9] and other developing African countries [10-22], with a preponderance of Burkitt's lymphomas, followed by retinoblastoma, nephroblastoma and soft tissue sarcomas as the commonest malignancies, and a low relative frequency of leukaemias and intracranial tumours [9-22]. This is in contrast to the situation in Europe and North America, with their high leukaemia and intracranial tumour frequency ratios, and low lymphoma frequency ratio. However, a report of frequency rates of childhood cancers in Namibia, a developing African country, showed an intermediate or mixed picture with tumours of the central nervous system occurring most commonly (18%), followed by renal tumours (14%), leukaemia (12%) and lymphoma (11.5%)[23]. One obvious departure though, is the second position of brain tumours in this paediatric malignancy mortality series, compared to the low relative frequency of intracranial tumours in most paediatric malignancy morbidity series from developing African counties [9-22], with the exception of Namibia[23]. The reasons for these differences require further investigation, though the high position of brain tumours in this mortality series may be as a result of its relatively higher case mortality rate in Ghana, and thus its possible over-representation in this mortality series.

## Conclusion

The patterns observed from this study, despite the obvious limitation of not being population-based, provide reliable information on cancer mortality patterns in Ghana. Whether the above observed patterns actually reflect parallel patterns in cancer morbidity and mortality in the general Ghanaian population will have to await further studies from the just established population-based cancer registry in Accra, Ghana.

## Abbreviations

Korle-Bu Teaching Hospital, KBTH;

Standard Deviation, SD;

ASCAR, Age-Standardized Cancer Ratio;

Confidence Interval, CI;

Relative Frequency, RF;

International Agency for Research on Cancer, IARC.

## Competing interests

The author(s) declare that they have no competing interests.

## Authors' contributions

EKW conceived and designed the study, provided guidance to all aspects of this study, and was the senior author. HBA was responsible for data collection and data entry. Both authors did the quality assessment of the data, data analysis, and data preparation. Both authors collaborated intensely on all aspects of the preparation of the manuscript, and approved the final manuscript.

## Pre-publication history

The pre-publication history for this paper can be accessed here:



## Figures and Tables

**Table 1:** Number of cases by age group and summary frequency rates in females

SITE	ALL AGES	0–14	15–24	25–34	35–44	45–54	55–64	≥ 65	RF %	ASCAR
BREAST	283	0	4	19	89	82	53	36	17.1	17.24
HAMATOPOIETIC ORGANS	300	96	46	34	29	35	32	28	18.2	14.69
LIVER	180	1	10	40	26	30	36	37	10.9	10.97
GALLBLADDER	48	0	0	1	3	6	9	29	2.9	3.84
PANCREAS	73	1	0	2	6	9	21	34	4.3	5.68
OESOPHAGUS	5	0	0	1	0	2	0	2	0.3	0.34
STOMACH	60	0	0	3	2	9	13	33	3.6	4.76
COLON & RECTUM	64	0	0	5	8	14	12	25	3.9	4.59
ANUS	1	0	0	0	0	1	0	0	0.1	0.04
CERVIX	122*	0	0	5	24	27	36	29	7.4	8.47
ENDOMETRIUM	63	0	6	13	9	14	12	9	3.8	3.75
OVARY	100	3	7	7	23	18	24	18	6.0	6.32
BRAIN	77	19	11	14	9	8	7	9	4.7	3.76
NASOPHARYNX	14	3	5	1	0	4	1	0	0.8	0.71
LARYNX	2	0	0	0	1	1	0	0	0.1	0.10
LUNG	37	1	2	5	4	8	5	12	2.2	2.43
BONE	21	6	4	5	2	3	1	0	1.3	0.88
KIDNEY	39	16	1	1	8	4	5	4	2.4	1.90
EYE	10	4	0	3	0	1	1	1	0.6	0.45
BLADDER	27	0	0	0	5	6	6	10	1.6	1.98
SOFT TISSUE	28	5	3	2	8	3	0	7	1.7	1.46
OTHER	97	11	7	10	13	15	18	23	5.9	5.67
ALL SITES	1651	166	106	171	269	300	292	346	100	100

* Age not stated for 1 case

**Table 2:** Number of cases by age group and summary frequency rates in males

SITE	ALL AGES	0–14	15–24	25–34	35–44	45–54	55–64	≥65	RF %	ASCAR
BREAST	12	0	0	1	2	0	3	6	0.6	0.66
HAEMATOPOIETIC ORGANS	417	126	72	73	45	39	26	36	20.8	15.57
LIVER	428	10	19	82	98	96	65	58	21.3	21.15
GALLBLADDER	12	0	1	0	1	3	2	5	0.6	0.70
PANCREAS	91*	2	6	4	3	17	23	35	4.5	5.22
OESOPHAGUS	39	0	0	0	5	6	15	13	1.9	2.42
STOMACH	126**	1	1	7	7	21	30	57	6.3	7.26
COLON & RECTUM	53	0	3	4	6	8	11	21	2.6	2.95
ANUS	6	0	0	0	1	2	1	2	0.3	0.36
BRAIN	67	18	9	7	11	6	7	9	3.3	2.78
NASOPHARYNX	20	2	3	4	1	6	2	2	1.0	0.93
LARYNX	45*	0	0	0	2	9	14	19	2.2	2.75
LUNG	78	1	2	6	5	18	22	24	3.9	4.56
BONE	34	5	10	6	5	3	3	2	1.7	1.35
PROSTATE	286	0	0	0	9	21	74	182	14.2	17.35
KIDNEY	41	18	5	3	1	2	7	5	2.0	1.63
BLADDER	91	1	2	7	12	23	14	32	4.5	5.07
EYE	14	11	2	0	0	1	0	0	0.7	0.40
SOFT TISSUE	19	4	0	4	4	3	3	1	0.9	0.83
OTHER	129	11	7	9	21	27	27	27	6.4	6.36
ALL SITES	2008	210	142	217	239	311	349	536	100	100

*Age not stated for 1 case.

** Age not stated for 2 cases
